# The Effects of Cyclooxygenase Inhibitors on the Brain Inflammatory Response Following Traumatic Brain Injury in Rats

**Published:** 2012

**Authors:** Zakieh keshavarzi, Mohammad Khaksari, Zohre Razmi, Ava Soltani Hekmat, Vida Naderi, Sima Rostami

**Affiliations:** 1 *North Khorasan University of Medical Sciences, Bojnord, Iran *; 2 *Physiology Research Centre, Kerman University of Medical Sciences, Kerman, Iran*; 3 *Kerman University of Medical Sciences, Kerman, Iran*

**Keywords:** Brain injury, Celecoxib, Ibuprofen, Interleukin-10

## Abstract

**Objective(s):**

Cytokines such as IL-1β are involved in inflammatory responses. This study evaluated the role of two different kinds of drugs (ibuprofen and celecoxib) on brain IL-10 and IL-1β after traumatic brain injury (TBI) in male rats.

**Materials and Methods:**

Rats were assigned into 6 groups: intact, sham, TBI, and treated rats with vehicle, celecoxib or iboprophen. Cytokine concentrations were quantified by ELISA kits.

**Results:**

Groups showed no significant difference in brain IL-10 either after TBI induction or after treatment with ibuprofen or celecoxib. Serum IL-10 in vehicle or ibuprofen treated animals was lower than in sham groups (*P*< 0.01). Brain IL-1β decreased after treatment by ibuprofen or celecoxib (*P*< 0.001). There was no statistical difference in serum IL-1β in TBI and intact. Serum IL-1β significantly decreased in rats that received celecoxib compared to TBI group (*P*< 0.01).

**Conclusion:**

Based on our study IL-1β can decrease through both cyclooxygenase 1 (COX-1) and COX-2 pathway but serum IL-1β can decrease only by COX-2 pathway.

## Introduction

Traumatic brain injury (TBI), a signiﬁcant health trouble, represents a potentially catastrophic weakening medical emergency with poor prediction and long-term disability. Each year in the US at least 1.4 million people request medical aid for a TBI, of which about 50 000 die and, 235 000 are hospitalized (1). 

Pro-inflammatory cytokines such as IL-1β and TNF-α are involved in ischemia/hypoxia and trauma-induced brain injury and they are the important mediators of the systemic host response to infection (2). 

It has been proposed that cyclooxygenase-2 (COX-2) inhibition is responsible for the therapeutic effects of non-steroidal anti-inflammatory drugs (NSAIDs), while COX-1 inhibition causes the gastrointestinal and renal side effects (3). These isoforms were named ‘constitutive’COX-1 and ‘inducible’ COX-2. COX-1 catalyzes the construction of cytoprotective prostaglandins (PGs) in thrombocytes, vascular endothelium, stomach mucosa, kidneys, pancreas, Langerhans islets and brain. As a result of studies focused on reduction of the adverse effects of NSAIDs, selective COX-2 inhibitors, such as celecoxib and rofecoxib, have been developed. Selective inhibitors of COX-2 are drugs whose therapeutic effects are as strong as conventional NSAIDs but which lead to fewer side effects. They possess analgesic, antipyretic and anti-inflammatory effects as potent as traditional anti-inflammatory drugs. Celecoxib and rofecoxib inhibit COX-2 375 800 times more strongly than COX-1, respectively (4).

In the present study, we evaluated the effects of COX-2 selective inhibitor (celecoxib) or non-selective COX inhibitor (iboprophen) on the levels of IL-10 and IL-1β of brain after traumatic brain injury in the male rats. 

## Materials and Methods

***Animals***

Male Wistar rats (250 to 300 g) aged 12 weeks were housed in temperature and humidity controlled animal quarters with a 12 hr light/dark cycle. All procedures were approved in accordance with the guidelines of the National Institutes of Health on the care and use of animals surgery.

***Experimental groups***

The rats were randomly divided into six groups (n= 7 in each group): 1) intact group: the animals that were not given any drugs; 2) sham group: rats were sham surgically, but without actual induction of TBI; 3) TBI group: intact rats were injured using the traumatic brain injury device; 4) vehicle group; rats were gavaged with 2 ml of normal saline; 5) celecoxib group; rats were gavaged with celecoxib (10 mg/kg) 6) iboprophen group; rats were gavaged with iboprophen (10 mg/kg). Rats of treatment groups received gavages at 1 hr before the surgery. The serum samples were harvested 24 hr after induction of trauma.

***Induction of TBI***

The TBI was moderate and diffused using the Marmarou method. The work process of the TBI induction device was as follows: a 250 g weight was dropped from a 2 meter height on the head of the anesthetized rat (5). 

***Measurement of brain cytokines***

Brain was weighed and homogenized in T-PERTM tissue protein extraction reagent with 0.5% Triton-100, 150 mM NaCl, 50 mM tris, and protease inhibitor cocktail. Following homogenization, the supernatant was collected as homogenate. The concentration of the cytokines was quantified as picogram of antigen per milliliter by ELISA kits (6). 

***Statistical analysis***

Software SPSS 11.5 was used in the statistical analysis. Each parameter was expressed as mean±SEM, and was interpreted through one-way ANOVA analysis of variance. The level of significance was *P*< 0.05.

## Results

***Brain level of IL-10 following treatment***

There was no significant difference in brain IL-10 level between different groups either after TBI induction or after treatment with ibuprofen or celecoxib (not shown).

***Serum level of IL-10 following treatment***

**Figure 1:**
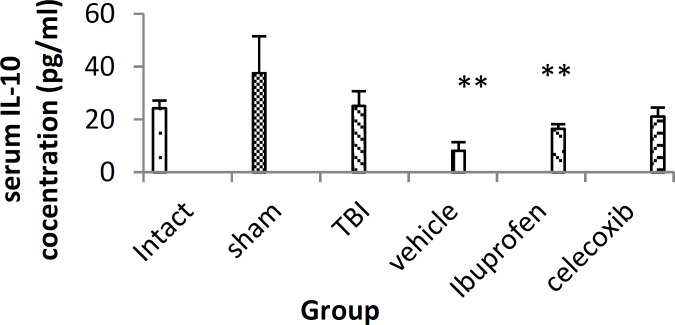
Serum IL-10 level in different groups (n= 7 in each group) after traumatic brain injury. TBI: Traumatic brain injury. Data are presented as mean±SEM.

TBI does not have any effect on the serum level of IL-10 compared to intact group. Injured animals treated with either vehicle (8.25±3.1 pg/ml, *P*< 0.01) or ibuprofen (16.57±1.73 pg/ml, *P*< 0.01) had lower level of IL-10 compared to sham (37.6±13.8 pg/ml) animal (Figure 1).

***Brain level of IL-1β following treatment***

**Figure 2:**
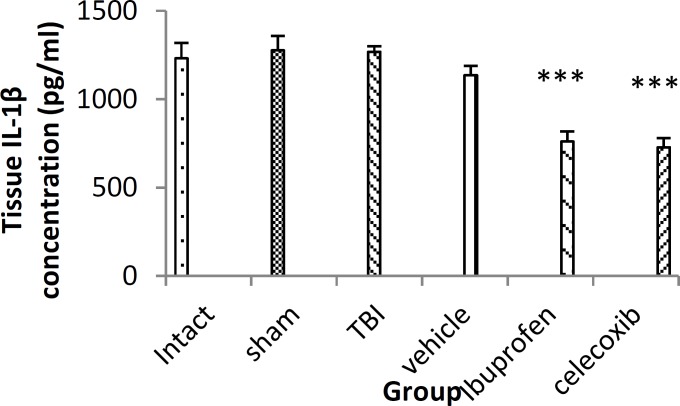
Brain IL-1β level in different groups (n=7 in each group) after traumatic brain injury. TBI: Traumatic brain injury. Data are presented as mean±SEM

Brain level of IL-1β does not change after TBI in male rats. In ibuprofen (760±57.11pg/ml) and celecoxib-treated groups (727±51 pg/ml), the brain level of IL-1β significantly decreased compared to vehicle group (1134 ± 53 pg/ml) (*P*< 0.001) (Figure 2). 

***Serum level of IL-1β following treatment***

The level of IL-1β significantly decreased in rats that received celecoxib (19.16±4.05) compared to TBI group (*P*< 0.01) (not shown).

**: *P*< 0.01, vehicle and Ibuprofen vs. sham group

## Discussion

Acute administration of ibuprofen has been observed to diminish the neuronal injury and infarct volume in ischemia models. Gopez *et al* showed that DFU improves functional recovery and reduces cell death and inflammation when administered systemically before or after TBI. In addition, this COX2 inhibitor attenuates injury-induced prostaglandin production in the brain, shifting arachidonic acid metabolism toward potentially neuroprotective eicosanoids (7). 

In the present study we observed that the serum levels of IL-1β have not changed after treatment by ibuprofen. This is consistent with other reporting that the IL-1β production was generally unaffected by ibuprofen (2).

On the other hand, pretreatment with ibuprofen and celecoxib significantly decreased brain IL1- β levels in our study. Celecoxib may decrease inflammation and brain edema with perihematomal cell death. COX-2 is rapidly induced in inflamed tissues, and its reactive products are accountable for many cytotoxic properties of inflammation. Because, COX-2 inhibition is at least part of the celecoxib's mechanism, these results propose that inhibition of COX-2 could have beneficial effects by altering the cascade of pathogenic processes in the experimental intracranial hemorrhage rat model (8).

IL-1ß mediates inflammatory responses after ischemic brain injury. In fact, IL1- β is an essential factor in postischemic brain damage (9). Thus, celecoxib and ibuprofen may exert some of their effects through decreasing IL-1ß.

The reports about the effect of ibuprofen on the cytokine production are controversial. Some investigators have reported an increased proinflammatory cytokine secretion, while others have found that it was reduced or unchanged. In the present study, pretreatment with ibuprofen significantly decreased serum levels of interlukin 10, but it had no effect on the brain IL-10 level (2). Because PGE2 is a potent inducer of IL-10 (10), inhibition of PGE2 by ibuprofen may play a role in decreasing of serum of IL-10. Celecoxib, a COX-2 selective inhibitor, also decreased serum IL-10. It was shown that, inhibition of centrally produced PGE2 by NSAIDs leads to an increase in the neuroinflammatory response (11).

Selective inhibitors of the inducible COX-2 isoforms such as celecoxib have led to contradictory results in experimental TBI (12). Decrease of the serum IL-10 by ibuprofen may explain some of these controversies about NSAIDs. 

## Conclusions

The applied weight was not enough for induction of changes in IL-10 and IL-1β levels. It seems that brain IL-1β levels can be minimized by both COX-1 and COX-2 pathways but serum IL-1β can decrease only by COX-2 pathway.
